# Synthesized Mammography: Clinical Evidence, Appearance, and Implementation

**DOI:** 10.3390/diagnostics8020022

**Published:** 2018-04-04

**Authors:** Melissa A. Durand

**Affiliations:** Department of Radiology, Yale University School of Medicine, New Haven, CT 06412, USA; melissa.durand@yale.edu; Tel.:+1-203-200-5590

**Keywords:** digital breast tomosynthesis, synthesized mammography, screening mammography, breast cancer detection, radiation dose

## Abstract

Digital breast tomosynthesis (DBT) has improved conventional mammography by increasing cancer detection while reducing recall rates. However, these benefits come at the cost of increased radiation dose. Synthesized mammography (s2D) has been developed to provide the advantages of DBT with nearly half the radiation dose. Since its F.D.A. approval, multiple studies have evaluated the clinical performance of s2D. In clinical practice, s2D images are not identical to conventional 2D images and are designed for interpretation with DBT as a complement. This article reviews the present literature to assess whether s2D is a practical alternative to conventional 2D, addresses the differences in mammographic appearance of findings, and provides suggestions for implementation into clinical practice.

## 1. Introduction

Digital breast tomosynthesis (DBT) has been heralded as an advancement of mammography by virtue of its significant improvements on performance outcomes. It is distinct from supplemental screening tools such as ultrasound and magnetic resonance imaging (MRI) as its use significantly increases cancer detection while simultaneously reducing recall rates. Single site and multi-site studies have validated DBT’s potential to benefit all women, regardless of age or breast density. In addition, long-term studies are beginning to reflect that the improvements seen with DBT are not bound to prevalent screening, but persist on incident screening. All this has made DBT increasingly requisite at many institutions, both community and academic [1,2,3,4,5,6,7,8,9,10,11].

The 2011 F.D.A. approval of DBT specified its performance in conjunction with 2D full-field digital mammography (FFDM): a 2D+DBT combined exam [12]. While still below federal radiation safety limits of 3 mGy per view, a two-view combined exam is however, approximately double the dose of the 2D FFDM exam alone [13]. Furthermore, this precludes women with implants or large breasts requiring tiling, from receiving DBT, as their screening exams already include additional views. In addition, though the patient may remain in the same compression, the DBT and conventional 2D exams are acquired sequentially, with the addition of DBT adding 3.7–25 s onto the conventional 2D exam, depending on vendor [14,15,16]. 

Synthesized mammography (s2D) was created in response to these challenges, with the goal of affording patients the benefits of tomosynthesis, minus the additional radiation and acquisition time and first gained United States Food and Drug Administration (FDA) approval in 2013 [17]. To evaluate s2D+DBT as a valid clinical tool, the synthesized mammography literature is reviewed here and appraised in accordance with known screening criteria. Implementation suggestions and case examples are also provided herein to ease transition for radiologists considering s2D+DBT as a replacement for 2D+DBT.

## 2. Technical Aspects of Synthesized Mammography

As of this writing, there are three vendors with approval for synthesized mammography in the US and Europe: Hologic, Inc. (C-view, Marlborough, MA, USA), GE Healthcare (V-Preview, Chicago, IL, USA), and Siemens (Insight, Munich, Germany). To create the synthesized 2D image, the DBT data set is collapsed into a single slice, similar to the maximum intensity projection (MIP) image in magnetic resonance imaging. Since the s2D image is generated from the DBT dataset, this obviates the additional radiation dose of the conventional 2D exposure. This also means s2D can be made from any view in which DBT is acquired, including spot compression views. As well, the additional time to acquire the 2D FFDM image is eliminated. In turn, this decreases the time patients remain in compression, which can reduce patient motion and may improve efficiency by increasing patient throughput. While s2D is performed without separate compression or acquisition, compression and breath hold are very important, as whatever affects the tomosynthesis images will affect the synthesized mammogram. Finally, the s2D image is labeled to differentiate it from a conventional 2D image [18].

s2D was not designed to be a carbon copy of 2D FFDM; the design goal was to offset the limitations of FFDM, while providing a useful 2D guide. The processing algorithm used to create s2D weights line structures like architectural distortions and spiculations, as well as high contrast features like calcifications, more than background fat and fibroglandular tissue. This increases their conspicuity, especially in denser regions of the breasts, where feature visibility might be obscured by superimposed tissue in FFDM [18]. 

In addition, as the s2D image is created from the DBT dataset, vendor differences in DBT acquisition results in vendor-specific DBT images and subsequently varying synthesized results. For example, GE utilizes a step and shoot system and adaptive statistical iterative reconstruction. Nine equal dose projection images are obtained over a 25-angular degree range. When the X ray beam is perpendicular to the detector, a central projection is acquired; the V-preview s2D image is reconstructed from this central projection by incorporating information from the other 8 projections [19]. For Siemens, 25 projection images are obtained over a 50 degree angular range, followed by enhanced multiple parameter iterative reconstruction, from which the Insight 2D image is created [20]. In the Hologic system, 15 projection images are obtained over 15 degree angular range followed by filtered backprojection reconstruction. Unlike GE and Siemens, pixel binning is employed which combines a cluster of pixels into a single pixel and can reduce scan time. However since the s2D image is generated from lower resolution tomosynthesis data, the end result is lower spatial resolution than 2D FFDM. While the GE and Siemens systems do not employ pixel binning, signal to noise ratios are also reduced in their projection images and the subsequent synthesized images are lower resolution relative to their respective 2D FFDM images. As such, objects with lower contrast may be harder to differentiate from background noise [21]. 

In a recent study comparing phantom image quality of s2D to 2D, Nelson et al. demonstrated that s2D has lower resolution at 5 line pairs/mm compared to 2D at 11 line pairs/mm. Indeed when held to ACR scoring criteria, the 2D FFDM phantom outscored the s2D phantom [22]. However, the reconstructed slices can have resolutions of approximately 100 microns, which coupled with high-resolution detectors and specially designed filters, should permit adequate visualization of microcalcifications [18]. As well, of foremost importance is that s2D is not a standalone exam. Unlike 2D, it should not be interpreted without the tomosynthesis complement. 

## 3. Purpose of the 2D Image

When trying to minimize acquisition and interpretation times, one might question whether the 2D image is really necessary; would tomosynthesis images be sufficient? The 2D image is a way to globally assess the breast. It provides a means to readily gauge breast density, evaluate symmetry, and consider interval changes from prior non-tomosynthesis exams. In addition, as calcifications may be distributed over a few DBT slices, it can allow better appreciation of these fine structures. Although maximal efficiency is always a goal, it is equally prudent to balance efficiency and quality. Hence, the utility of the 2D image remains.

## 4. Early s2D Prototype Reader Studies

Prior to FDA approval, one of the earliest s2D studies was a 2012 reader study involving ten radiologists and a prototype unit, in which 114 s2+DBT exams were compared to 2D+DBT. Gur et al. found that while non-cancer recalls were similar in both groups, sensitivity was higher with conventional 2D+DBT compared to s2D+DBT (0.826 vs. 0.772, *p* = 0.017), and all readers in the synthesized group missed 16 clusters of microcalcifications [23]. 

However, another multi-reader study evaluated s2D+DBT performance compared to 2D FFDM via crossover reading of a cancer-enriched set of 302 cases. The first half of the 2D FFDM cases and the second half of the s2D+DBT cases were read, followed by the first half of the s2D+DBT cases and second half of the 2D FFDM cases, with a 1-month wash-out period. Radiologists’ performance was compared between 2D and s2D+DBT using the area under the receiver operating characteristic (ROC) curve and outcome metrics. Results showed a superior ROC performance curve and reduced non-cancer false positives in the s2D+DBT group compared to 2D FFDM. These performance improvements were maintained in spite of significantly lower dose estimates for the s2D+DBT exam compared to 2D+DBT (1.45 mGy s2D+DBT, 2.65 mGy 2D+DBT, 1.2 mGy 2D FFDM alone). These results were part of this vendor’s submission for the first FDA approval of synthesized mammography in 2013 [24]. 

In 2014, Skaane et al. reported results of a large study comparing s2D+DBT and 2D+DBT 12,621 cases. Still utilizing a prototype s2D, the initial results demonstrated fewer false positives in the synthesized group (4.6%; 582/12621 vs. 5.3%; 670/12621. However cancer detection was lower in the synthesized group (7.4 per 1000 vs. 8.0 per 1000). After a software upgrade, false positives remained lower in the synthesized group, and cancer detection essentially equalized: one fewer cancer was found in the synthesized group (94 vs. 95 cancers in 12,270 cases). Importantly, they concluded that s2D+DBT could result in a 45% reduction in radiation dose while maintaining comparable performance to 2D+DBT [25]. 

The same year, Zuley et al. were the first to compare s2D alone to 2D FFDM; they also compared s2D+DBT to 2D+DBT. This study of 123 cases included 9 cancers shown as calcifications only. Unlike results from the Gur et al.’s earlier prototype study in which malignant microcalcifications were missed, sensitivity in this study was similar between the 2 groups (88% 2D+DBT, 86% s2D+DBT). This may reflect the difference in later prototype iterations used versus the Gur study [23]. Interestingly, although s2D is not used alone in the clinical setting, Zuley et al. found s2D alone (AUC 0.894) and 2D FFDM (AUC 0.889) performed similarly, with nonsignificant differences in areas under the curve. Similar performance was also noted for s2D+DBT (AUC 0.916) and 2D+DBT (0.939) [26]. Thus, despite initial skepticism, progressive prototype versions established a foundation from which clinical use could grow. 

## 5. Synthesized Mammography Clinical Studies

### 5.1. Recall Rates

In 2016, Bernardi et al. published results from their prospective population screening study, STORM-2, in which parallel, independent double reading of 9672 exams was performed using a commercially available tomosynthesis system capable of synthesized mammography. Readers A and B prospectively assessed cases in 2D, followed by 2D+DBT; readers C and D assessed cases in s2D followed by s2D+DBT. There was no significant difference in recall rate for s2D+DBT (4.45%) and 2D+DBT (3.97%). Adding DBT increased recall rates by 0.5–1% over 2D FFDM (2D 3.42%). This increase may have occurred because all recalls were included, after reading any part of the exam, even s2D alone, with no arbitration of discordant double reads. As stated earlier, in clinical use, s2D is always read in conjunction with the DBT component. Notably, the recall rates of all groups in the STORM-2 were still very low compared to typical US recall rates (optimal < 12%). Furthermore, screening practices in the STORM-2 study differ from typical North American standards (excluding women <49 years of age and employing biennial screening frequency) [27]. 

Subsequent North American studies have consistently demonstrated reduced recall rates with synthesized mammography. Zuckerman et al.’s retrospective study compared 15,571 patients screened with 2D+DBT and 5366 patients screened with s2D+DBT. Despite more baseline screening exams in the synthesized group, recall rates were significantly reduced in the s2D+DBT group compared to 2D+DBT (7.1% vs. 8.8% respectively, *p* < 0.001) [28]. 

Among 5342 2D+DBT cases and 14,980 s2D+DBT cases Ambinder et al. presented comparable recall rates between the 2 groups (7% 2D+DBT vs. 7.2% s2D+DBT, *p* = 0.66, with no differences in recall rates of masses, calcifications, asymmetries, or architectural distortions [29].

Aujero et al.’s large retrospective study compared 32,076 2D exams, 30,561 2D+DBT exams, and 16,173 s2D+DBT exams and confirmed that recall rates remain significantly reduced with s2D+DBT (s2D+DBT 4.3%, 2D+DBT 5.8%, 2D FFDM 8.7%), even after adjustment for age, race, and breast density [30]. 

Freer et al.’s multivariate retrospective institutional analysis of 9525 s2D+DBT, 1019 2D+DBT, and 21,435 2D FFDM screening exams further established that recall rate reductions could be maintained with s2D. This study notably controlled for effects of age, density, prior mammograms, and interpreting radiologist, thereby demonstrating the power of synthesized technology itself without confounding factors. Significant recall reductions were seen in the s2D+DBT cohort compared to 2D FFDM (5.52% vs. 7.83%, *p* < 0.001). There was a non-significant decrease compared to 2D+DBT (6.39%), however given the small size of the 2D+DBT cohort, this group was limited by selection bias and comparison to this group was underpowered to detect a true change [31]. 

These studies substantiate the claim that the benefits of low recall rates seen with digital breast tomosynthesis implementation can be upheld with synthesized mammography, while simultaneously decreasing radiation dose. 

### 5.2. Cancer Detection Rates

STORM-2 demonstrated a significant increase in cancer detection rate (CDR) when DBT was added to 2D FFDM (8.5 per 1000 2D+DBT, 6.3 per 1000 2D FFDM). This improvement was maintained and actually slightly increased with s2D+DBT (8.8 per 1000). Incremental CDRs over 2D alone were also similar between s2D+DBT (+2.5 per 1000) and 2D+DBT (+2.2 per 1000). The most significant improvements in cancer detection were seen in women <60 years of age and women with dense breasts. As biennial screening is the standard in European screening programs, the CDRs reported in STORM-2 are higher than typical North American practices [27]. 

In North American studies, the use of synthesized mammography has shown consistent trends towards increased CDRs. To that point, a study from Johns Hopkins showed a non-significant increase in CDR to 5.27 per 1000 in their s2D+DBT group compared to 4.97 per 1000 in their 2D+DBT group, *p* = 0.81 [29]. 

Aujero et al.’s results showed a slight increase in cancer detection from 2D FFDM (5.3 per 1000) to 2D+DBT (6.4 per 1000), which was maintained with s2D+DBT (6.1 per 1000). Notably, the percentage of invasive cancers detected with s2D+DBT was significantly higher than 2D and 2D+DBT (s2D+DBT 76.5%, 2D 61%, 2D+DBT 61.3%; *p* < 0.01), without a loss in in situ cancer detection. This was thought to reflect a learning curve of using DBT, as the s2D+DBT studies were interpreted after some years of experience with DBT [30]. 

Freer et al.’s study also showed cancer detection rates were maintained in the s2D+DBT group (5.4 per 1000) compared to 2D FFDM (5.0 per 1000), even after controlling for the use of prior mammograms, patient age, breast density, and radiologist. No difference in invasive and in situ CDRs or percentage of node negative cancers was observed [31].

Thus, audit parameters for cancer detection have been preserved in the face of false positive reductions and reduced radiation dose, with synthesized mammography use. 

### 5.3. Biopsy Rates and Positive Predictive Values

With s2D, biopsy rates have also significantly decreased (1.3% s2D+DBT, 2% 2D+DBT, *p* = 0.001), well below the national recommended biopsy rate of 3% [28]. Additionally, positive predictive values from recall (PPV1) and from biopsy (PPV3) have been markedly increased with s2D+DBT (PPV1: 14.3% with s2D+DBT, 6% with 2D FFDM; PPV3: 40.8% with s2D+DBT, 22.2% with 2D FFDM) [30]. This may in part reflect increased experience with DBT. Likewise, significant increases in PPV1 with s2D+DBT relative to 2D FFDM, (9.1% s2D+DBT, 6.2% 2D FFDM, *p* = 0.02) and a trend towards higher PPV2, and PPV3, have been demonstrated. In light of reduced recall rates, this suggests higher accuracy with synthesized mammography [31]. 

### 5.4. Sensitivity and Specificity

Results of the TOMMY trial, a multi-reader, multi-center retrospective study of 7060 cases showed that both sensitivity and specificity of s2D+DBT (88%; 72%) was similar to 2D+DBT (89%; 70%). Sensitivity was also similar compared to 2D alone (87%), however specificity was significantly higher in both s2D+DBT and 2D+DBT compared to 2D FFDM (57%, *p* < 0.001). This increase in specificity was seen in all subgroups of breast density, mammographic feature, and age [32]. 

In a retrospective reader study of 250 lesions enriched with malignancies, Mariscotti et al. compared the performance of s2D alone compared to 2D FFDM. While it is acknowledged that s2D is not meant as a stand-alone replacement for 2D, but as a complement to DBT, the design of this study allowed for direct comparison of these two technologies on the same study group. The readers interpreted studies with 2D FFDM, followed by s2D in a different order, with a 1-month washout period. In contrast to the TOMMY trial, overall, sensitivity was significantly higher with s2D compared to 2D FFDM (92% vs. 87%, *p* = 0.02) while specificity was similar between the 2 modalities (s2D 60%, 2D FFDM 62%, *p* = 0.21) [33]. 

Choi et al.’s retrospective reader study of 107 stage T1 invasive cancers and 107 negative cases also compared s2D to 2D FFDM. There was no difference in sensitivity between the 2 modalities (s2D 62.6–71.0%; 2D FFDM 60.7–71.0%). One reader showed significantly higher specificity with s2D (s2D 84.1% vs. 2D FFDM 72.9%, *p* = 0.02); the other 2 readers showed non-statistically significant increases in specificity (s2D 91.6–96.3% vs. 2D FFDM 88.8–94.4%) [34].

These results indicate comparable, and even improved, diagnostic performance of s2D+DBT versus 2D FFDM for breast cancer detection. 

### 5.5. Radiation Dose

Most tomosynthesis systems use the same target material, tungsten (W), for DBT and FFDM. However different filters are selected by the automatic exposure control (AEC) depending on breast thickness, vendor, and whether DBT or FFDM is performed (e.g., rhodium (Rh), silver (Ag), aluminum (Al)). Tube voltages are also variable, and can range from 25–33 kV_p_ for FFDM and 26–40 kV_p_ for DBT. For FFDM, the AEC increases kV_p_ and the number of X-ray photons or tube load (mAs) proportionally as breast thickness increases, to keep the contrast of the X-ray beam high enough to produce a high quality image. For DBT, the increase in mAs is more gradual as kV_p_ is increased. High contrast projection images are not needed for DBT as the reconstruction algorithm clears superimposed tissue, thus recovering contrast. However, to get adequate signal-to-noise ratio for high quality reconstructed images, greater kV_p_ and filtration are needed to create more penetrating X-ray beams. All of this affects mean glandular dose (MGD). In addition, MGD depends on breast absorption properties such as thickness and density, which can be affected by compression force and pressure. It increases with greater breast thickness or lesser compression, and decreases with breast density [35]. 

For a given breast density or thickness, MGD is typically higher for DBT than for FFDM. Gennaro et al. found a 38% increase in radiation dose per view with DBT compared to FFDM, similar to the dose difference between screen-film and digital mammography [35]. Lang et al. reported per-view MGDs of 1.2 mGy for FFDM and 1.6 mGy for DBT for a 33% dose increase [4]. And Skaane et al. reported a 23% dose increase, with 1.58 mGy for FFDM and 1.95 mGy for DBT [2]. As such, exams performed in the combined 2D FFDM+DBT mode are at least double the radiation dose of 2D FFDM alone. While the combined exams should remain within the 3 mGy per view safety limits required by the F.D.A., dose reduction strategies are a laudable goal. 

In keeping with prototype studies [25], clinical studies with synthesized mammography report significant reductions in radiation dose. Utilizing a custom dose-tracking system to extract factors such as breast thickness, kV_p_, mAs, filter, and exposure from the Digital Communications in Medicine (DICOM) header, Zuckerman et al. calculated average glandular dose per study. They found the average glandular dose per study was 39% lower for s2D+DBT compared to 2D+DBT (4.88 mGy vs. 7.97 mGy, *p* < 0.001) [28]. Similarly, STORM-2 results reported a 42% reduction in average glandular dose with s2D+DBT compared to the combined exam [27]. As shown above, this dose reduction is possible without a loss in performance outcomes; indeed in many instances, performance has improved. 

## 6. Synthesized Mammography Appearance

### 6.1. Breast Density

Breast density assessment is important, not only from the standpoint of its inherent risk, but as increasingly many states having breast density notification laws, this can affect supplementary screening options. Consistency in breast density interpretation thus has important downstream effects.

In a retrospective reader study of 309 cases and three readers, Alshafeiy et al. compared 2D FFDM and s2D breast density classification. They showed near perfect agreement (κ = 0.83) in two-category breast density classification and substantial consensus agreement (κ = 0.73) using the four-category BIRADS scale. However, by individual reader, variability in density categorization was present, with one reader more likely to assign a dense categorization on 2D FFDM and another more likely to assign a dense categorization on s2D [36]. 

Qualitative differences among breast density categories, especially at the extremes of breast density (predominantly fatty and extremely dense) have also been demonstrated [37]. Two other clinical studies have also shown that breast density was subjectively assessed as less on s2D compared to 2D FFDM [28,30].

Recently, Conant et al. evaluated automated assessment of density for s2D and 2D. They found strong correlation for percentage density between s2D and 2D (*r* = 0.92), though the more dense the tissue, the more disagreement emerged. Unlike the subjective density assessments in prior two studies above, automated density estimates in this study were 1.7% higher on s2D [38]. 

**Figure 1:**
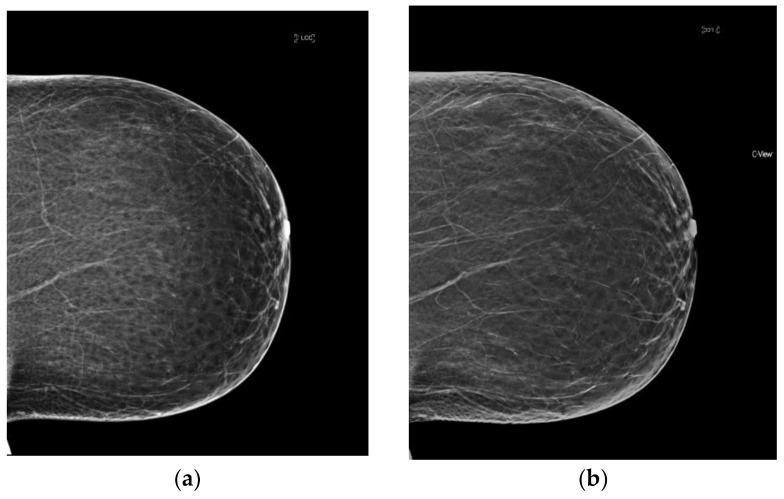

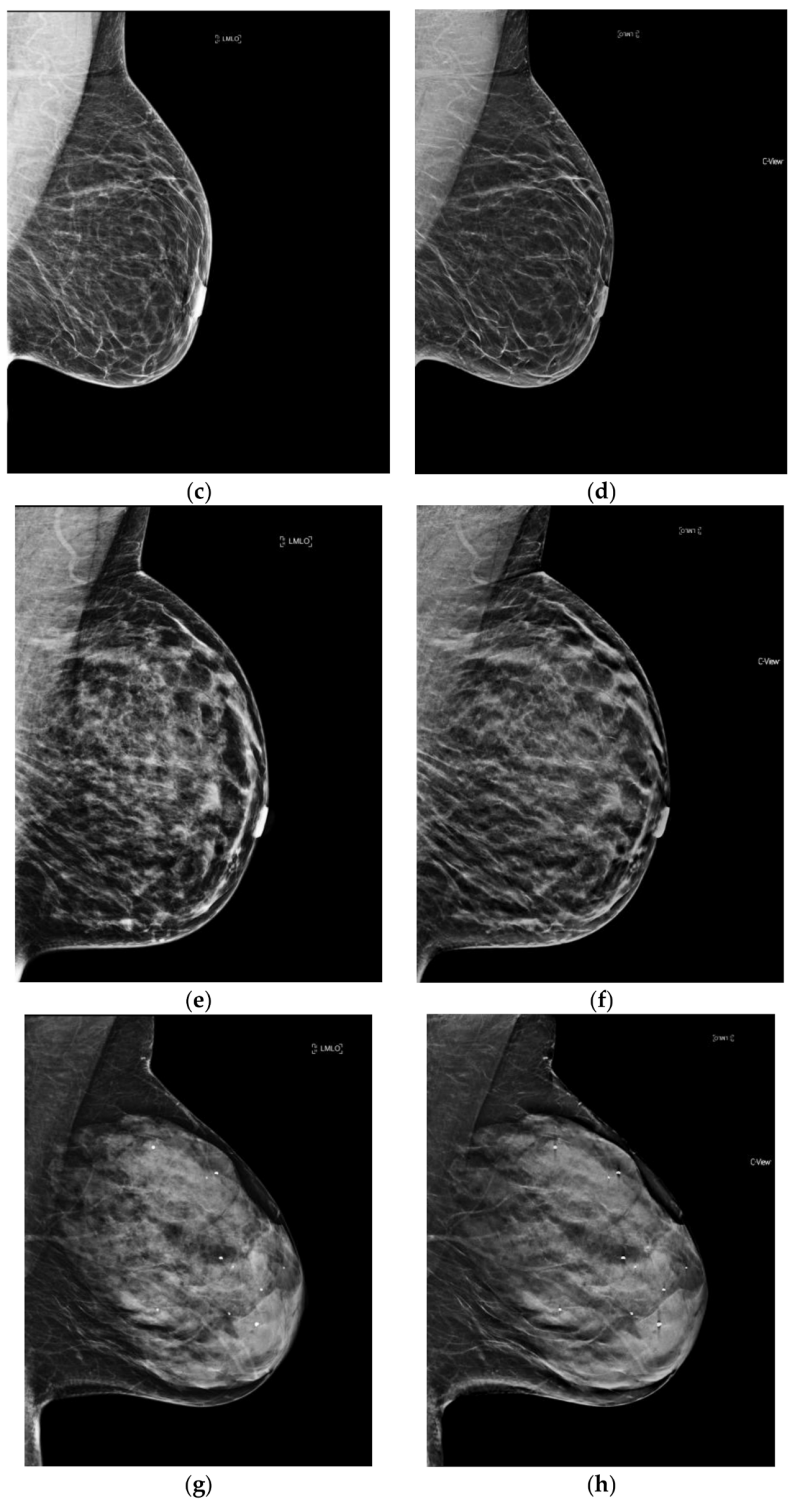
The four categories of breast density on conventional 2D FFDM and synthesized mammography. (**a**) Predominantly fatty breast 2D FFDM (**b**) Predominantly fatty breast s2D FFDM (**c**) Scattered fibroglandular breast 2D FFDM (**d**) Scattered fibroglandular breast s2D (**e**) Heterogeneously dense breast 2D FFDM (**f**) Heterogeneously dense breast s2D (**g**) Extremely dense breast 2D FFDM (**h**) Extremely dense breast s2D.

Of note, with synthesized mammography, the recall rate (RR) of women with dense breasts has been shown to approach that of 2D FFDM for women with non-dense breasts (RR dense breasts with s2D+DBT 7.11 vs. RR non-dense breasts with 2D FFDM 7.34%) [31]. Additional studies to subanalyze the effect of breast density on performance outcomes with synthesized mammography are needed. (Figure 1).

### 6.2. Calcifications

The processing algorithm to generate an s2D image enhances objects above a certain density threshold on s2D. In a phantom study, s2D provided better visualization of medium and large calcifications however smaller calcifications, especially fine calcifications with contrast similar appearance to background noise were harder to see [22]. On the contrary, in clinical use, a prospective review of 1206 screening exams found synthesized mammography to have significantly better conspicuity for calcifications compared to 2D FFDM (*p* < 0.001) [39]. 

This enhancement can also lead to parenchymal densities or ligaments falsely appearing as calcifications. Indeed, pseudo-calcifications are a recognized synthesized mammography artifact. When questioning the verity of a calcification finding, it is helpful to confirm identification in both planes, as well as on tomosynthesis images. Pseudo-calcifications will not be identifiable on both planes and may align with ligaments or vessels on tomosynthesis. Indeed, although reduced recalls for calcifications were seen with s2D+DBT compared to 2D FFDM, Freer et al. found calcification recalls were increased relative to 2D+DBT, which may reflect the effect of the pseudo-calcification artifact [31]. In contrast, Zuckerman et al. showed recall rates were lower for calcified lesions on s2D+DBT compared to 2D+DBT [28]. 

**Figure 2:**
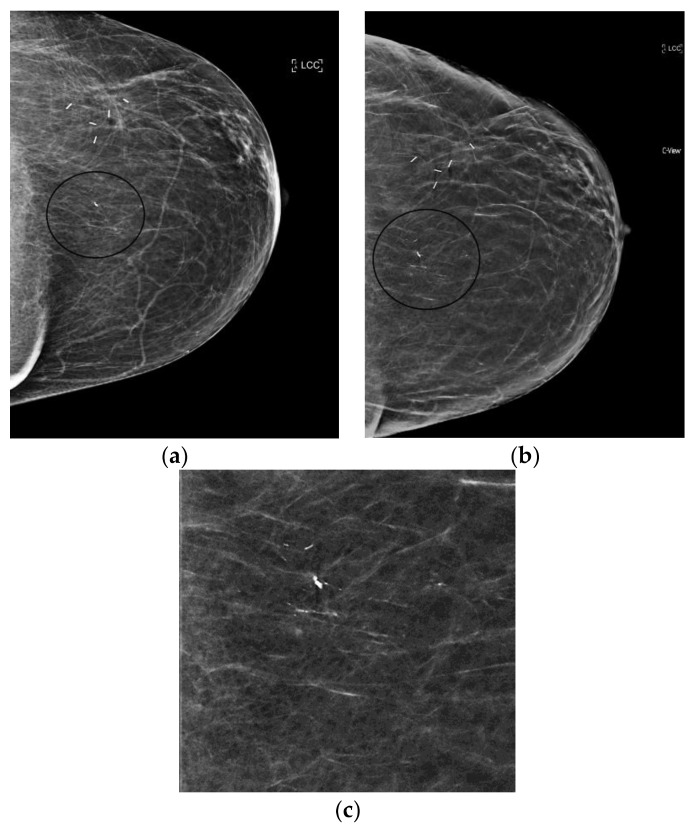
Linear, heterogeneous calcifications (circled in black) best seen on synthesized mammography images (grade 2 ductal carcinoma in situ). (**a**) 2D FFDM (**b**) s2D (**c**) s2D zoom.

s2D+DBT was inferior to 2D+DBT and 2D FFDM alone for detection of microcalcifications and DCIS in the 11-20mm range in the TOMMY trial [32]. And specificity for calcifications has been shown to be lower compared to masses and asymmetries for both s2D and 2D FFDM [33]. Despite this, in situ cancer detection was not affected by decreased recall rates for calcifications in Freer’s study [31]. Furthermore, Choi et al. showed similar performance of s2D and FFDM in detection of calcified and non-calcified stage T1 cancers [34]. How synthesized mammography definitively affects the rate of cancers detected as calcifications remains to be fully validated. (Figure 2).

### 6.3. Architectural Distortion

**Figure 3:**
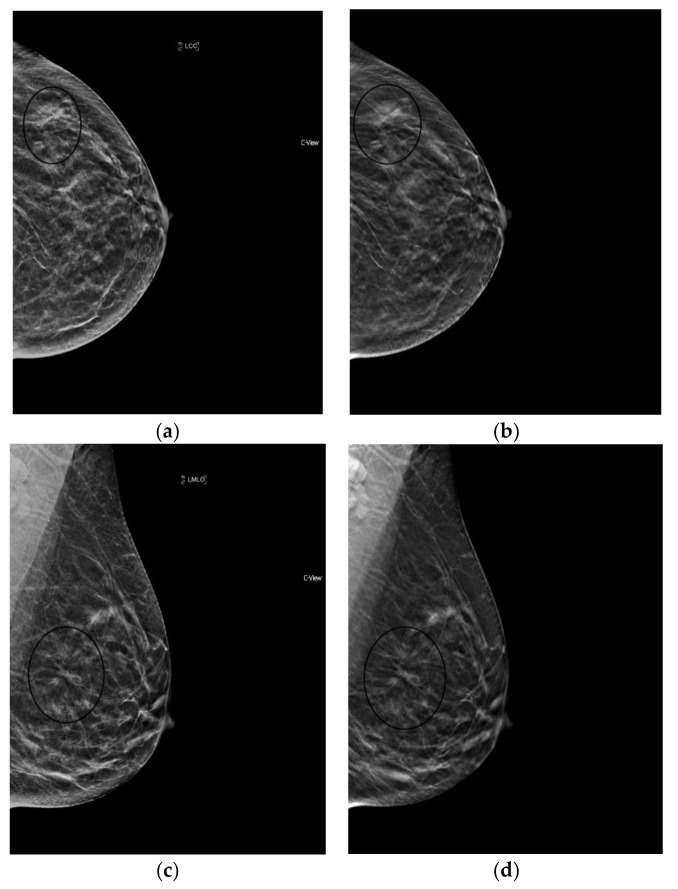
Architectural Distortion (circled in black) detected on screening mammogram with synthesized mammography and confirmed on tomosynthesis images (slices shown here), permitting confident Breast Imaging Reporting and Data System (BIRADS) 0 assessment. After diagnostic work-up and biopsy, pathology confirmed well-differentiated invasive ductal carcinoma, ER/PR+, Her2-, with grade 1 ductal carcinoma in situ. (**a**) s2D CC (**b**) DBT slice CC (**c**) s2D mediolateral oblique (**d**) DBT slice mediolateral oblique.

The reconstruction algorithm’s enhancement of line structures can also emphasize the appearance of architectural distortions (AD). Taken together with the ability of tomosynthesis to clear away superimposed tissue, s2D+DBT can permit confident identification of subtle cancers presenting as AD. (Figure 3). 

Giess et al. showed architectural distortions were significantly more conspicuous on synthesized mammography compared to FFDM (*p* < 0.001) [39]. Indeed, Mariscotti et al. found poor concordance in classification of AD between s2D and 2D FFDM (κ = 0.36), with six malignant ADs missed on 2D FFDM [33]. As s2D is derived from the DBT dataset, it may retain more information on tissue structure from the multiple projection images compared to 2D FFDM, emphasizing mammographic findings like AD. 

Freer et al. reported no significant change in recall rates for AD with synthesized mammography [31]. This may reflect that AD characterization is best defined by tomosynthesis images, rather than by 2D or synthesized 2D. 

It is important to note that normal ligaments may also be enhanced and present as possible distortions. As with calcifications, confirmation in both planes and with tomosynthesis can avoid false positive recalls. Careful diagnostic work-up and wariness of distortions that “spot away” remains prudent, given the high probability of malignancy of ADs. Additional studies could explore whether use of s2D increases detection of benign radial scars/complex sclerosing lesions, which may also present as AD. 

### 6.4. Masses and Asymmetries

**Figure 4:**
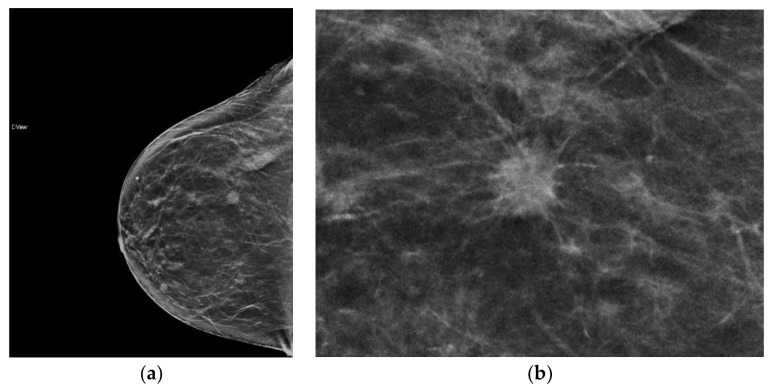
Mass, readily detected on synthesized mammography, with clear depiction of spiculated margin. Invasive ductal carcinoma, moderately differentiated, ER/PR+, Her2−. (**a**) s2D RCC mass (**b**) s2D RCC zoom.

**Figure 5:**
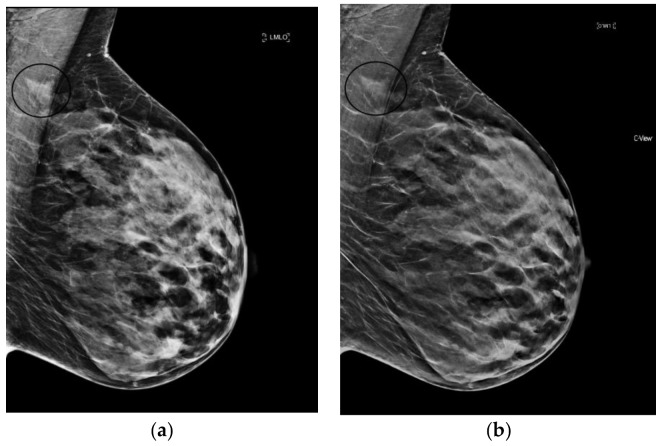
Indeterminate asymmetry seen on 2D FFDM is less prominent on synthesized mammography and resolved on DBT slices; no recall was necessary. (**a**) 2D FFDM (**b**) s2D.

With 2D FFDM, overlapping tissue may obscure the margins of masses and create asymmetrical areas of tissue. One of the benefits of tomosynthesis is the improvement in margin analysis of masses as this tissue is cleared away. This information is passed onto the synthesized image as it is created from the DBT data set, which can lead to clearer conspicuity of masses and their margins (Figure 4). Potentially false positive asymmetries can also be resolved on tomosynthesis. Since s2D is always read in conjunction with DBT, these benefits remain when synthesized mammography is used. (Figure 5).

When stratified by mammographic finding, recall rates were shown to be lower with s2D+DBT for calcified lesions and asymmetries, with no significant change in recalls of architectural distortion or masses. This result could be because AD and masses are best resolved on DBT images, which were utilized in both the synthesized and FFDM groups [28].

### 6.5. Artifacts

**Figure 6:**
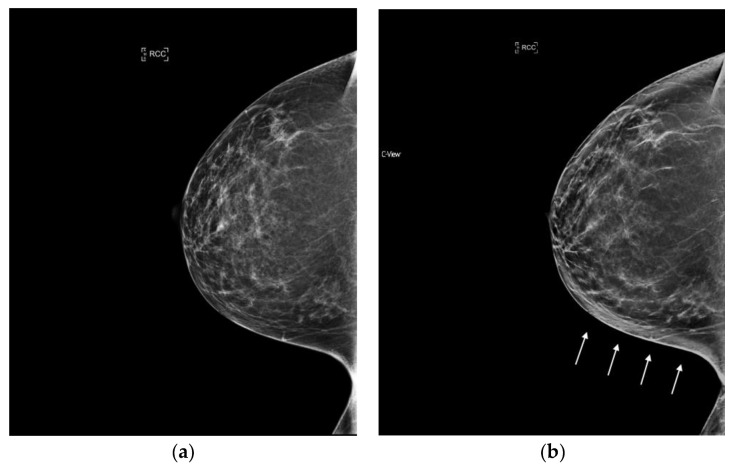
Bright band artifact (indicated by arrows) can be seen along the curvature of the medial breast on synthesized mammography. (**a**) 2D FFDM (no artifact) (**b**) s2D with Bright band artifact.

**Figure 7:**
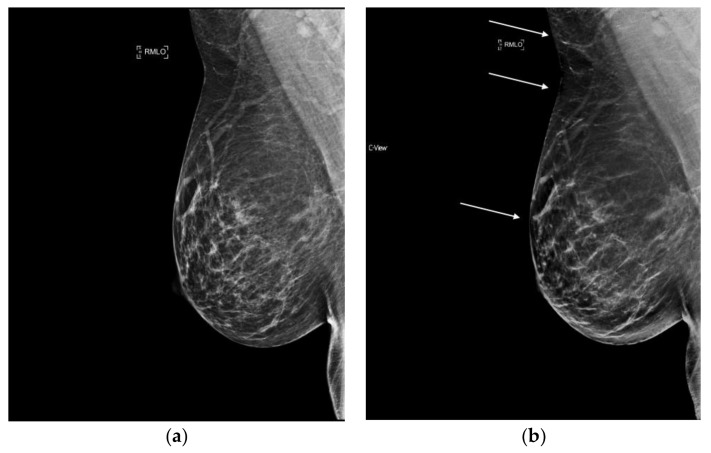
Loss of skin resolution or “skin burnout” (indicated by arrows) can be seen on synthesized mammography. (**a**) 2D FFDM (no artifact) (**b**) s2D with skin burnout artifact.

Awareness of a few artifacts unique to s2D can ease familiarization with synthesized images. A bright-band artifact may be observed with s2D as a layer of brighter tissue, which follows the curvature of the breast at the subcutaneous level. (Figure 6). Typically this area contains fatty tissue and lesion detection can still be made readily. Additionally, especially in very dense or very thick breasts, because higher radiation dose is needed, the detector can get saturated at the skin line and cause loss of resolution of the skin line. (Figure 7). 

**Figure 8:**
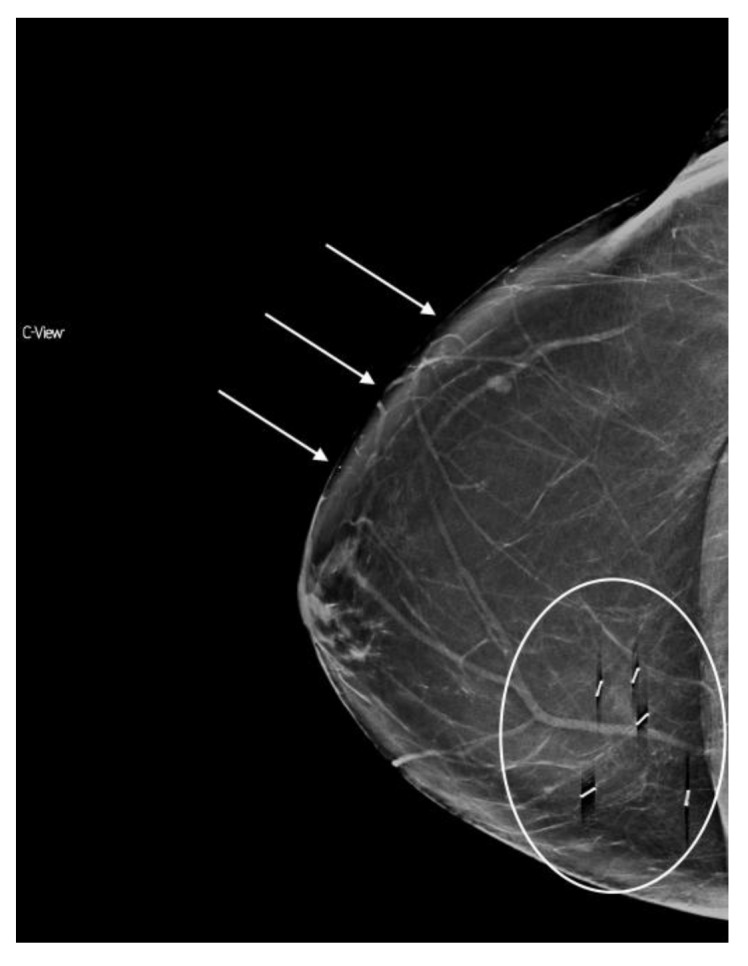
“Slinky”/shadowing clip artifact (circled) and skin burnout (arrows) on synthesized mammography.

Surgical clips, biopsy markers, and dense calcifications may present with a slinky artifact, which results in dark linear bands below the object. Shadowing below the object may also occur due to the edge-enhancement filters used during s2D reconstruction. (Figure 8). An algorithm has been developed that can eliminate the “slinky” artifact and decrease shadowing, which may be applied by the technologist after image acquisition. It is important to differentiate these artifacts from the “zig-zag” appearance that can be created by patient motion. Since the s2D image is created from the DBT dataset, any motion during the DBT acquisition will affect the s2D image. If motion is present, the artifact seen with metal clips or calcifications will not be completely vertical, but instead will have a “zig-zag” appearance. Movement of the inframammary fold on the tomosynthesis projection images can also be a clue to patient motion [37,40].

## 7. Synthesized Mammography Implementation

In a 2017 survey of breast radiologists from the Radiological Society of North America database, 1092 responses reported that the majority of DBT users still rely on the 2D FFDM image: 286 (40.7%) use 2D FFDM+DBT, 246 (35%) use 2D FFDM+DBT+s2D, and just 170 (24.2%) use s2D+DBT. However, of those using 2D+DBT or 2D+DBT+s2D, 58.2% planned to transition to s2D in place of 2D FFDM in the future [41]. 

To facilitate the transition to synthesized mammography, an overlap period of at least a few months, during which patients are screened with 2D FFDM as well as s2D + DBT, can be beneficial. A hanging protocol designed to view s2D+DBT images before the 2D FFDM encourages readers to make decisions based on the synthesized and tomosynthesis images, increases reader comfort with the s2D images, while maintaining the availability of the conventional 2D image for verification. Eventually, as readers grow accustomed to the s2D images, 2D FFDM can be phased out. 

Commercially available computer-aided detection (CAD) exists for digital breast tomosynthesis. In a cancer-enriched reader study, 20 readers interpreted 120 cases with CAD and 120 cases without CAD and were evaluated for performance and reading time. In this study, DBT was only available for the MLO images and the CAD system used was designed to detect soft-tissue densities (AD, masses, asymmetries); it did not detect calcifications. When CAD was used, this was displayed as an enhanced synthetic MLO image; no outlines or marks indicated the presence of the lesions. Mean AUC increased from 0.841 without CAD to 0.850 with CAD (*p* < 0.01), with reading times decreasing for 19/20 readers and overall 29.2% faster reading time with CAD [42]. Another study tested CAD performance using a retrospectively randomly selected dataset of 175 screening and diagnostic patients, which included 124 malignant lesions and 52 normal exams. This CAD system did permit detection of soft-tissue densities as well as calcifications and was available for CC and MLO images; DBT was also available on all CC and MLO images. High per-lesion sensitivity for cancers was observed with CAD (89%; 99/111), with an acceptable false positive rate (2.7 per breast view) [43]. Larger studies are needed to assess whether CAD can positively affect early cancer detection and consistently decrease reading times. 

## 8. Conclusions

Since its approval for clinical use in 2013, multiple studies have demonstrated that use of synthesized mammography can decrease radiation dose by nearly half, while maintaining the performance benefits of DBT over FFDM. Although lower resolution than FFDM, with s2D, cancer detection is preserved and the risks of missing a low-density finding is outweighed by this benefit as well as the decreased dose. Differences in the appearance of the s2D image exist, and this may require an adjustment period. Future research avenues remain to optimize use of s2D. Given the present literature field, s2D+DBT is expected to change DBT practice patterns, in favor of increasing implementation of s2D+DBT as a replacement for conventional 2D+DBT.
